# Ecology of Phlebotomine Sand Flies in the Rural Community of Mont Rolland (Thiès Region, Senegal): Area of Transmission of Canine Leishmaniasis

**DOI:** 10.1371/journal.pone.0014773

**Published:** 2011-03-21

**Authors:** Massila W. Senghor, Malick N. Faye, Babacar Faye, Karamoko Diarra, Eric Elguero, Oumar Gaye, Anne-Laure Bañuls, Abdoul A. Niang

**Affiliations:** 1 Laboratoire de Zoologie des Invertébrés Terrestres, Institut Fondamental d'Afrique Noire Cheikh Anta Diop, Université Cheikh Anta Diop, Dakar, Sénégal; 2 Département de Biologie Animale, Faculté des Sciences, Université Cheikh Anta Diop, Dakar, Sénégal; 3 Service de Parasitologie-Mycologie Médicale, Université Cheikh Anta Diop, Dakar, Sénégal; 4 Laboratoire MIVEGEC (UMR IRD 224-CNRS 5290-Université Montpellier 1), Montpellier, France; University of Bristol, United Kingdom

## Abstract

**Background:**

Different epidemiological studies previously indicated that canine leishmaniasis is present in the region of Thiès (Senegal). However, the risks to human health, the transmission cycle and particularly the implicated vectors are unknown.

**Methodology/Principal Findings:**

To improve our knowledge on the population of phlebotomine sand flies and the potential vectors of canine leishmaniasis, sand flies were collected using sticky traps, light traps and indoor spraying method using pyrethroid insecticides in 16 villages of the rural community of Mont Rolland (Thiès region) between March and July 2005. The 3788 phlebotomine sand flies we collected (2044 males, 1744 females) were distributed among 9 species of which 2 belonged to the genus *Phlebotomus*: *P. duboscqi* (vector of cutaneous leishmaniasis in Senegal) and *P. rodhaini*. The other species belonged to the genus *Sergentomyia*: *S. adleri, S. clydei, S. antennata, S. buxtoni, S. dubia, S. schwetzi* and *S. magna*. The number of individuals and the species composition differed according to the type of trap, suggesting variable, species-related degrees of endophily or exophily. The two species of the genus *Phlebotomus* were markedly under-represented in comparison to the species of the genus *Sergentomyia*. This study also shows a heterogeneous spatial distribution within the rural community that could be explained by the different ecosystems and particularly the soil characteristics of this community. Finally, the presence of the *S. dubia* species appeared to be significantly associated with canine leishmaniasis seroprevalence in dogs.

**Conclusions/Significance:**

Our data allow us to hypothesize that the species of the genus *Sergentomyia* and particularly the species *S. dubia* and *S. schwetzi* might be capable of transmitting canine leishmaniasis. These results challenge the dogma that leishmaniasis is exclusively transmitted by species of the genus *Phlebotomus* in the Old World. This hypothesis should be more thoroughly evaluated.

## Introduction

In the Thiès region, canine leishmaniasis was recorded for the first time in 1970 by Ranque and Bussiéras [1]. Many studies have shown that it is endemic in this area [1]-[3], whereas human visceral leishmaniasis has never been observed. A single case was described in Gambia, a country that is almost completely surrounded by Senegal [4], [5].

The most recent study was carried out by Bâ *et al.* [6] with the aim of investigating the population dynamics of phlebotomine sand flies and making an inventory of the viruses that they transmit. In this occasion, 0.38% *Phlebotomus* and 99.62% *Sergentomyia* were collected.

However, a recent epidemiological survey conducted by medical and veterinary doctors has shown that the parasite was still circulating hyper-endemically and that canine leishmaniasis was still rampant in the area. Indeed, more than 30% of dogs were infected and 30% of humans were found to be seropositive for leishmaniasis [7]. At present, the biological cycle of canine leishmaniasis in Senegal has not been elucidated yet and particularly the vector responsible of the transmission is not known.

Therefore, following these recent epidemiological data on humans and dogs and due to the limited knowledge about the vector(s) involved, we started an extensive entomological study in the rural community of Mont Rolland. The main objective was to evaluate the ecology of phlebotomine sand flies in this area in order to better target the potential vectors of leishmaniasis. To this aim, we inventoried the sand fly fauna in the rural community of Mont Rolland and studied their spatial distribution according to ecosystems and *Leishmania* seroprevalence in dogs that was determined in a parallel survey [7]. This first analysis has allowed improving the knowledge about the phlebotomine fauna and will be valuable for further studies on the cycle of the canine leishmaniasis in the Thiès region.

## Methods

### The study area

**Figure 1:**
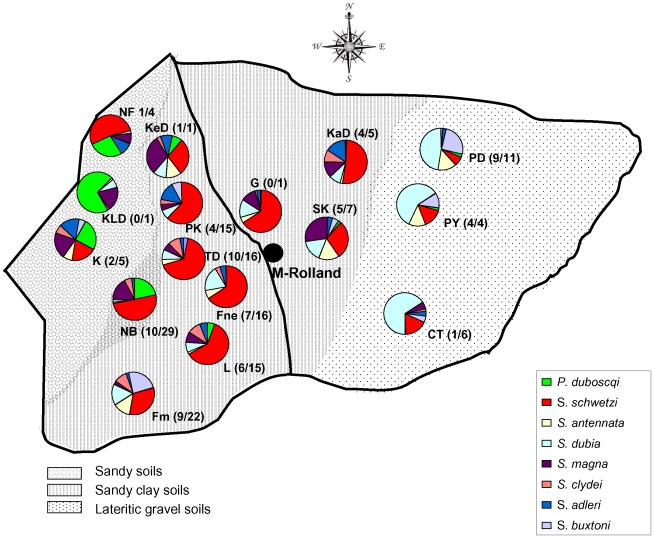
Distribution of sand fly species and leishmaniasis seropositive dogs in the rural community of Mont Rolland. The map shows the rural community of Mont Rolland (the villages are represented by their initials), the distribution of the different species of phlebotomine sand flies in each village and, in between brackets, the number of seropositive dogs out of the total number of tested dogs in each village [7].

The rural community of Mont Rolland is located in the Thiès region (West Senegal), at about 15 Km north of this city and between latitudes 14°55′–14°56′N and longitudes 16°50–16°55′W. The climate is tropical of the type typical of the Soudan-Sahel region. The rainy season lasts in general from June to October. The annual amount of precipitation for this area is comprised within 500 and 650 mm. The mean annual temperature is 26.7°C. The lowest temperatures are recorded during the dry season, with a minimum of 24.4°C and the most elevated ones during the rainy season with a maximum of 29.2°C. Hygrometry presents seasonal and daily variations. The maximum is about 90% during the second half of the night in the rainy season, while the minimum is about 25% at the end of the dry season during the day. Pedological and phytogeographical data, collected by the Department of Geography and Statistics of Dakar, have shown that, in the west part of the community, soils are ferruginous and mainly sandy with rare vegetation; in the east part, soils are characterized by lateritic gravel with a vegetation of thorn trees, whereas at the centre of the community there are sandy clay soils with shrubby vegetation (Fig. 1 and Table 1).

**Table 1:** Ecosystems, number of traps and number of sand flies in each trap in the different villages.

Villages	Eco	CDCNo.T (No.flies)	STNo. T (No. flies)	IRSNo. T (No. flies)	Total	Mean No.flies/T
G	SC	14 (175)	39 (361)	14 (21)	557	23.26
KaD	SC	14 (18)	21 (163)	14 (35)	216	11.55
Fm	SC	6 (27)	113 (883)	14 (18)	928	11.03
SK	SC	8 (40)	15 (11)	9 (12)	63	8.73
TD	SC	11 (24)	29 (128)	8 (10)	162	8.66
NB	S/SC	14 (13)	92 (525)	14 (16)	554	8.03
L	SC	14 (4)	70 (283)	14 (9)	296	4.97
PD	LG	14 (8)	106 (186)	14 (23)	217	3.97
PY	LG	14 (13)	66 (44)	14 (32)	89	3.88
PK	SC	14 (3)	21 (66)	14 (4)	73	3.64
K	S	14 (6)	54 (152)	14 (5)	163	3.60
Fne	SC	14 (26)	71 (70)	14 (7)	103	3.34
KeD	SC	14 (2)	63 (131)	14 (0)	133	2.22
CT	LG	14 (0)	74 (131)	14 (2)	133	1.91
KLD	S	14 (2)	70 (47)	14 (3)	52	1.03
NF	S	3 (0)	54 (49)	3 (0)	49	0.91

The table presents the number of phlebotomine sand flies collected in each of the 16 villages:

Guidieur (G), Khaye Diagal (KaD), Fouloum (Fm), Sambaye Karang (SK), Twin Djassa (TD), Ndiaye Bopp (NB), Loukhouss (L), Pallo Dial (PD), Pallo Youga (PY), Paham Kouye (PK), Kémaye (K), Fouloune (Fne), Keur Daouda (KeD), Colobane Thiombane (CT), Keur Lat Diop (KLD) and Nguith Fall (NF); the different traps used [light traps (CDC), sticky traps (ST) and indoor residual spraying (IRS)]; the number of traps (No.T) and in bracket the number of sand flies caught **(**No.flies) in each village with that type of trap; the mean number of sand flies/trap (Mean No.flies/T). Eco, Ecosystem; SC, sandy clay soil; S, sandy soil; LG, lateritic gravel soil.

*The Ndiaye Bopp village presents intermediate ecological features between S and SC.

### Collection of phlebotomine sand flies

Sand flies were collected from March to July 2005 during the same 7 days/each month in the following 16 villages (Fig. 1): Colobane Thiombane (CT), Fouloum (Fm), Fouloune (Fne), Nguith Fall (NF), Guidieur (G), Keur Daouda (KeD), Keur Lat Diop (KLD), Kémaye (K), Khaye Diagal (KaD), Loukhouss (L), Ndiaye Bopp (NB), Paham Kouye (PK), Pallo Youga (PY), Pallo Dial (PD), Sambaye Karang (SK) and Twin Djassa (TD). Figure 1 shows the number of dogs that were seropositive for canine leishmaniasis out of the total number of dogs tested in each village during the veterinary study carried out by Faye et al. [7]

Three types of traps were used to capture the maximum number of specimens with different behaviours (endophilic, exophilic, etc…):

- **Sticky strips** (white paper sheets 20×20 cm coated with castor oil) were placed at the entrance of rodent burrows and other crevices (holes in termite nests, soil cracks, tree trunks) and collected the day after.
- CDC **light traps** were placed indoors or outdoors in proximity of houses (kennels, poultry houses, etc).

Sticky and CDC light traps were installed before sunset and retrieved the following morning. This time interval includes the periods of intense activity of sand flies: dusk and dawn.

- **Indoor sprayings,** with the insecticide “Yotox” (pyretrin is the active compound), were carried out in three rooms/village between 8 and 11 am. After spraying, rooms were kept closed for 7–12 minutes. Insects were collected on white sheets that had been previously put on the floor. Specimens were then carefully removed using a small brush and placed in tubes containing 70% alcohol.

### Insect handling

Phlebotomine sand flies caught on sticky straps and indoor spraying were removed and stored in 70% alcohol up to their identification. Insect collected using light traps were sorted out after cold anaesthesia and stored in 70% alcohol up to their identification.

Phlebotomine sand flies were mounted permanently on slides using Euparal or Canada balsam, after clearing in 20% potassium for two hours, washing in distilled water twice for 30 minutes, clearing in Hoyer medium modified according to Marc-André for 1 hour, dehydratation in 70% (30 minutes) and then 90% alcohol (30 minutes). Sand fly species were identified according to the morphological key features described by Abonnec using a photonic force microscope after drying the slides in an oven at 40°C for at least 48 hours [8].

### Statistical Analysis

To investigate the possible association between species distribution and ecosystems, a discriminant analysis was carried out using the type of ecosystem as a classification variable, and the number of captures per trap as explaining variables. Since the discriminant analysis can only help identifying variables (species) related to the classification in the different ecosystems, it gives no information on which species is related to which ecosystem. Hence, in a second analysis we studied each ecosystem independently. For each ecosystem, we carried out logistic regression analyses to identify the species that influenced the probability to be located in that ecosystem.

Finally, logistic regression analysis was employed to investigate which species abundances could explain the proportion of infected dogs. For this purpose, we used the data from the serological studies of canine *Leishmania* infection performed by Faye et al. [7]. All statistical analyses were performed using the R *Statistical Package* [9].

## Results

### Phlebotomine fauna

The three types of traps allowed collecting 3788 phlebotomine sand flies. The mean number of insects captured in each of the 16 villages of the rural community of Mont Rolland is presented in Table 1. The geographical distribution of phlebotomine sand flies in the villages was heterogeneous and ranged from 0.91 insects/trap in the village of Nguith Fall to 23.26 insects/trap in Guidieur (G).

The phlebotomine fauna (2 044 males and 1 744 females) included two genera, 5 sub-genera and 9 species among which 2 of the genus *Phlebotomus: Phlebotomus* (*Phlebotomus) duboscqi* Neveu-Lemaire, 1906 (vector of the cutaneous leishmaniasis in Senegal) and *Phlebotomus* (*Anaphlebotomus) rodhaini* Parrot, 1930; and 7 of the genus *Sergentomyia: Sergentomyia (Sintonius) adleri* Theodor, 1933, *Sergentomyia (Sintonius) clydei* (Sinton, 1928), *Sergentomyia (Sergentomyia) antennata* Newstead, 1912, *Sergentomyia (Sergentomyia) buxtoni* Theodor, 1933, *Sergentomyia (Sergentomyia) dubia* Parrot, Mornet and Cadenat, 1945, (vector of the gecko leishmaniasis in Senegal), *Sergentomyia (Sergentomyia) schwetzi* Adler, Theodor and Parrot, 1929, and *Sergentomyia (Parrotomyia) magna* Sinton, 1932.

The nine species of the genus *Sergentomyia* constituted 93.71% of the total capture of which four constituted over 75.5% of the collections: *S. schwetzi*, *S. dubia S. buxtoni* and *S. magna* (Table 2).

**Table 2:** Sand fly species caught in the Mont Rolland rural community.

Species	Males (%)	Females (%)	Total (%)
*P. duboscqi*	147 (7.18)	90 (5.17)	237 (6.26)
*P. rodhaini*	0 (0.00)	1 (0.05)	1 (0.03)
*S. schwetzi*	889 (43.43)	685 (39.35)	1574 (41.55)
*S. antennata*	105* (5.14)	111 (6.37)	216 (5.89)
*S. dubia*	231* (11.30)	243 (13.93)	474 (12.51)
*S. magna*	170 (8.30)	190 (10.91)	360 (9.50)
*S. clydei*	141 (6.89)	138 (7.93)	279 (7.37)
*S. adleri*	88 (4.30)	122 (7.01)	210 (5.55)
*S. buxtoni*	273 (13.33)	164 (9.42)	437 (11.54)
Total	2044 (54.04)	1745 (45.96)	3788 (100)

This table presents the nine species of sand flies caught in the Mont Rolland rural community, the number of females and males and their percentage in the whole population.

*Differently from females, the distinction between males of *S. dubia* and males of *S. antennata* was difficult. Therefore we calculated the ratio between *S. dubia* and *S. antennata* females (2.19) and then we used the same ratio to estimate the number of males in each of the two species.

### Species distribution relative to the type of capture

#### Sticky traps

The population (57% males and 43% females) caught with sticky traps in peri-domestic habitats represented 85.27% of the overall collection (Table 3). The four most common species were the same as in the global population: *S. schwetzi*, *S. buxtoni*, *S. dubia* and *S. magna*). The two species of *Phlebotomus* were poorly represented, particularly *P. rodhaini*. However, it is with this type of trap, we collected the highest number of *P. duboscqi* (91% of the overall collection for this species).

**Table 3:** Sand fly species collected in each type of traps.

	Sticky Traps			CDC Light Traps			Indoor Spraying		
species	M	F	Total (%)	M	F	Total (%)	M	F	Total (%)
*P. duboscqi*	140	75	215 (6.66)	5	10	15 (4.16)	2	5	7 (3.55)
*P. rodhaini*	0	1	1 (0.03)	0	0	0	0	0	0
*S. schwetzi*	777	520	1297 (40.09)	85	152	237 (65.65)	27	13	40 (20.30)
*S. antennata*	83	79	162 (4.98)	11	18	29 (7.76)	11	14	25 (12.69)
*S. dubia*	178	159	337 (10.65)	16	22	38 (11.08)	37	62	99 (50.25)
*S. magna*	147	158	305 (9.38)	18	19	37 (9.97)	5	13	18 (9.14)
*S. clydei*	140	136	276 (8.51)	0	1	1 (0.28)	1	1	2 (1.02)
*S. adleri*	88	119	207 (6.41)	0	3	3 (0.83)	0	0	0
*S. buxtoni*	268	162	430 (13.25)	1	0	1 (0.28)	4	2	6 (3.05)
**Total**	**1821**	**1409**	**3230**	**136**	**225**	**361**	**87**	**110**	**197**

The table presents the number of individual of each species (the number of males (M), females (F), total and percentage) collected in each type of trap (sticky traps; CDC light traps; Indoor Spraying).

#### Light traps

The light traps, which were settled out indoors or in proximity of houses, represented 9.53% of the overall collection. Females (62%) were predominant. The most common species were *S. schwetzi*, *S. dubia*, and *S. magna*. The other *Sergentomyia* species were rare or absent, and *P. duboscqi* was the only species of *Phlebotomus* caught (Table 3).

#### Indoor spraying

The capture by indoor spraying of pyrethroid insecticides represented 5.20% of the entire collection. Females (55.33%) were found to be predominant and *S. dubia* (vector of leishmaniasis of the gecko in Senegal) was the most abundant species, followed by *S. schwetzi*, *S. antennata* and then *S. magna* (Table 3). The other *Sergentomyia* species were rare or absent. *P. duboscqi* was not very common and *P. rodhaini* was absent (Table 3). *S. dubia* showed the most endophilic behaviour based on the indoors presence in majority of females with many fed (23) or gravid (39) individuals.

### Distribution of the species in the villages and relative to the ecosystems and dog seroprevalence

Species distribution was heterogeneous with differences according to the villages and also to the ecosystems. *S. schwetzi* was the most abundant and frequent species in most villages and particularly at the centre of the area under study (Fig. 1). The species *S. magna* was present in most of the villages. *S. buxtoni*, confined to termite nests, was collected mainly at Fouloum (Fm) at the entrance of the holes of a termite mound at the periphery of the village. It also represented an important part of the collection with sticky traps at Pallo Youga (PY) and Pallo Dial (PD) (Fig. 1). *S. dubia* was frequently captured in most of the villages, but predominated especially in three villages: Pallo Youga (PY), Pallo Dial (PD) and Colobane Thiombane (CT). *P. duboscqi* presented a high population size in four villages: Nguith Fall (NF), Keur Lat Diop (KLD), Kémaye (K) and Ndiaye Bopp (NB) (Fig. 1). The other species showed a relatively low prevalence in most of the villages without particular differences among the different areas.

**Figure 2:**
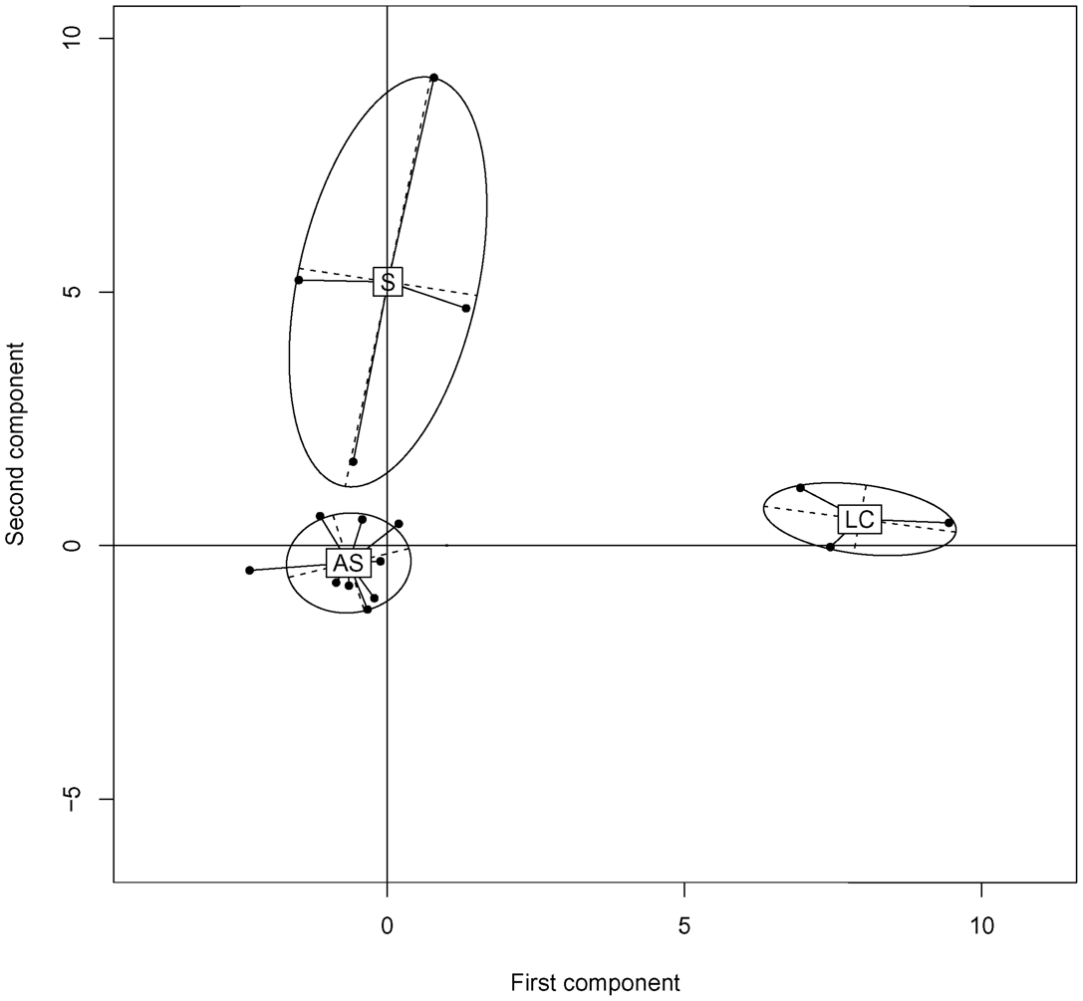
Discriminant analysis showing the relations between sand fly species and the three ecosystems. Each dot represents one village. Villages were classified in the ecosystem (LG, S and SC) they belong to. Sand fly captures were used to compute the coordinates of the dots (dimensionless units). Confidence ellipses materialized the variability of capture data across ecosystems. The Ndiaye Bopp (NB) village was excluded from this analysis because it is the only village with an intermediate ecosystem between S and SC (see Figure 1).

The study area could be subdivided into three ecosystems (Fig. 1): lateritic gravel soils with thorn trees (LG) in the east, sandy clay soils with bushy vegetation in the centre (SC), essentially sandy soils with rare vegetation in the west (S). Linear discriminant analysis (Fig. 2) showed that data of sand fly collection followed this ecosystem subdivision.

Logistic regression analysis allowed us to study the association between the frequency of each sand fly species capture and the ecosystem type. The strength of this association was measured using Odds-Ratios (OR): an OR lower than 1 indicated a negative association whereas an OR higher than 1 implied a positive association. Results are summarized in Table 4. *S. dubia* showed a significant positive association with the ecosystem LG and *P. duboscqi* with the ecosystem S. Conversely, they showed a significant negative association with the ecosystem SC. *S. schwetzi* and *S. antennata* were negatively associated with the environment LG.

**Table 4:** Ecosystems and sand fly captures.

	S ecosystem	SC ecosystem	LG ecosystem
species	OR (*P*-value)	OR (*P*-value)	OR (*P*-value)
PD	3.30 (0.0015) **	0.29 (0.0017) **	1.03 (0.88)
SA	0.98 (0.98)	2.62 (0.30)	0.39 (0.049) *
SS	1.04 (0.39)	1.07 (0.18)	0.89 (0.00003) ***
SD	0.86 (0.42)	0.59 (0.0047) **	1.96 (<10^−11^) ***
SM	0.87 (0.74)	0.93 (0.87)	1.24 (0.36)
SC	0.86 (0.19)	1.37 (0.55)	1.41 (0.20)
SA.1	1.27 (0.61)	1.06 (0.90)	0.74 (0.24)
SB	1.25 (0.58)	0.74 (0.48)	1.08 (0.73)

Odds ratios are computed by multiple logistic regression analysis (all species included). *P. duboscqi* (PD), *S. antennata* (SA), *S. schwetzi* (SS), *S. dubia* (SD), *S. magna* (SM), *S. clydei* (SC), *S*. *adleri* (SA.1), *S. buxtoni.* The indicated *P*-values result from likelihood ratio tests. Stars denote the degree of significance (* ≤5%, ** <1%, *** <0.1%).

The analysis by logistic regression of the sand fly captures and the dog seroprevalence is summarized in Table 5. *S. dubia* was found to be the unique species which is significantly associated with increased seroprevalence of leishmaniasis in dogs. The significance of the association of *S. antennata* with increased seroprevalence is dubious. Indeed, the very large variance of the effect estimate indicates that it was influenced by a small subset of data. *S. buxtoni* was associated with a reduction in seroprevalence.

**Table 5:** Leishmaniasis prevalence in dogs and sand fly captures.

Species	OR (95% CI)	*P*-value (LR test)
PD	4.13 (0.79–21.6)	0.088
SA	46.22 (1.38–1550.9)	0.031*
SS	0.75 (0.52–1.07)	0.12
SD	3.12 (1.16–8.39)	0.019*
SM	0.23 (0.037–1.44)	0.12
SC	2.87 (0.23–35.44)	0.41
SA.1	6.26 (0.67–58.4)	0.098
SB	0.072 (0.014–0.38)	0.0012**

Odds ratios are computed simultaneously using multiple logistic regression analysis (all species included), *P. duboscqi* (PD), *S. antennata*(SA), *S. schwetzi* (SS), *S. dubia* (SD), *S. magna* (SM), *S. clydei* (SC), *S*. *adleri* (SA.1), *S. buxtoni* (SB), *P*-values corresponding to likelihood ratio tests. (* ≤5%, ** <1%).

## Discussion

### Species identification

During this study in the rural community of Mont Rolland we captured nine of the thirty phlebotomine species inventoried in Senegal [10]-[12]. In this region, Bâ *et al.* [6] carried out sampling all year around and reported the presence of five additional species, *S.* (*Grassomyia*) *ghesquierei, S.* (*Grassomyia*) *inermis, S.* (*Grassomyia*) *squamipleuris, S.* (*Sintonius*) *affinis vorax* et *S.* (*Sergentomyia*) *distincta*. The absence of these species in our collection might be due to the fact that we did not explore their biotope [8], [13], [14]. Indeed, we only collected insects within and in the immediate surroundings of villages because we specifically intended to study the populations of phlebotomine sand flies that are in contact with humans and dogs (potential reservoir of visceral leishmaniasis), the other actors involved in the epidemiological cycle of canine leishmaniasis.

The predominance of the genus *Sergentomyia* (93.7%) in comparison to the genus *Phlebotomus* is in accordance with the works by Trouillet *et al.* [15] in the Ferlo (98.7%), Bâ *et al.* 12] at Kédougou (99.27%) and Bâ *et al.* [6] in the same locality (99.62%). The prevalence of *S. schwetzi* (41.55% of all captures) is in agreement with the works by Desjeux and Dedet; Blanchot *et al*. and Dedet *et al.* [16]-[18] in the focus of cutaneous leishmaniasis of Keur Moussa. Conversely, at Kédougou, which is more than 500 Km away from this area, Bâ *et al.* collected only 9% of *S. schwetzi* [12]. This species is largely widespread in Africa, south of the Sahara, and it is known for its polyvalent behaviour as it feeds on different animals including humans and dogs [8], [15]. In our study, three species showed abundances around 10%: *S. dubia* (13%), *S. buxtoni* (11.54%) and *S. magna* (9.5%). *S. dubia*, the vector of the gecko leishmaniasis in Senegal [19] is known for its strong ability of adaptation [8], [15].

The genus *Phlebotomus* was represented by two species: *P. duboscqi* (6.26%), the vector of human cutaneous leishmaniasis in Senegal, and a single specimen of *P. rodhaini* captured in the Loukhouss village. Bâ *et al.* [6] also collected a very small number of individuals of these two species (0.14% *P. duboscqi* and 0.24% *P. rodhaini*) while Ranque [20] reported the absence of *P. duboscqi* between July and August 1970 and July 1971. On the other hand, it was captured all year around in the cutaneous leishmaniasis focus of Keur-Moussa, which is located at about 27 km from the Mont Rolland region [16], [20]. Beside, our group previously obtained more than 30% of this species in this cutaneous leishmaniasis focus [21]. *P. rodhaini*, which has been suspected for long time to be involved in the transmission of leishmaniasis in Senegal, has always been collected in low proportion in the entire country and no individual of this species has been found to be infected by *Leishmania* parasites.

### Spatial distribution, ecosystem and seroprevalence

The mean number of phlebotomine sand flies varied hugely among the villages under study (from 23.26 insects per trap in Guidieur to only 0.91 in Nguith fall) and these differences do not seem to be linked to the distribution of seroprevalence in dogs.

On the other hand, our findings show that the specific spatial distribution of the different phlebotomine species can be explained by the three different ecosystems of this region (see Figs. 1 and 2). As example, *P. duboscqi* is mainly collected in the West side of this area. This indicates that, even in a limited area (here the community of Mont Rolland), the phlebotomine population can considerably vary according to the ecosystem. This feature should be taken into consideration when studying leishmaniasis, because these results suggest that vector species can be completely different from one area to the other, even if they are contiguous.

Sticky traps allowed to collect the highest number of individuals in each species [6], [10], [13], [15]. *S. schwetzi* was the dominant species captured by sticky traps (used peri-domestic) habitats and light traps (used indoors and in peri-domestic habitats), suggesting a more exophilic behaviour. Conversely, *S. dubia* was the species more frequently captured by insecticide spraying indoors, showing a more endophilic behaviour. Abonnenc also reported its frequent and abundant presence indoors [8]. *S. buxtoni*, a very exophilic species, which is more frequently confined to termite nests than to other biotopes [6], [15], [22], was indeed collected mainly in two termite mounds at Fouloum, where it represented 95% of the collection. It was rarely found indoors. We also observed differences according to capture methods in agreement with the literature [6], [10].

The differential composition in phlebotomine species according to the capture methods and thus the degree of endophily and exophily suggests that the transmission might be done by different vectors within a single area.

### Epidemiological hypothesis

These findings raise a major question: which species, based on their abundance, distribution and/or behaviour, could potentially play a role in the transmission of canine leishmaniasis in this area?

Previously published epidemiological studies show that the proportion of infected insects and thus capable of transmitting the disease is generally low [23]-[25]. Moreover, it is known that phlebotomine sand flies can usually fly only limited distance and this strongly affects the boundaries of their transmission areas [26]. The transmission of the disease in a given focus requires thus the coexistence of an adequate number of vectors around the parasite reservoir. Consequently, the risk is elevated around an infected individual, like in the case of a dog that lives in the neighbourhood of a phlebotomine-favourable niche, and gradually decreases when moving away from this source of parasites [27], [28]. In accordance with the literature, our working hypothesis was that the vector species (one or more) should belong to the genus *Phlebotomus.* Indeed, this genus is the only acknowledged vector of leishmaniasis in the Old Word [29]-[31]. However, only two species of this genus were collected at Mont Rolland, *P. duboscqi* and *P. rodhaini*. The rarity, particularly of *P. rodhaini,* the small population of *P. duboscqi* and its much localized presence in the west of the study area under study strongly suggest that these two species would not be involved in the transmission of canine leishmaniasis. Moreover, previous works have demonstrated that *P. duboscqi*, the main vector of the species *Leishmania major*, cannot transmit the species of the *L. donovani* complex [32]. In this study, all the other sand fly species that were captured around the habitats of dogs and humans belong to the genus *Sergentomyia*. These results suggest the possibility that certain species of *Sergentomyia* may play the role of vector. This conclusion challenges the dogma that leishmaniasis is exclusively transmitted by species of the genus *Phlebotomus* in the Old Word. The heterogeneous distribution of the phlebotomine species and the observation that canine leishmaniasis is rampant in the entire area under study suggest that different species could play the role of vector in a more or less prominent way according to the villages and the ecosystems. This hypothesis is strengthened by the significant association between the distribution of *S. dubia* and the seroprevalence in dogs. In this context our results suggest that *S. dubia* might be the main vector of canine leishmaniasis in the east part of the community. However, this does not mean that the other species are not involved in the transmission. Particularly, in most of the villages, *S. schwetzi* was the predominant species in peri-domestic habitats and thus around dogs and was ubiquitously distributed in the community. Therefore, it is also important to consider this species as one of the potential vectors. Other species, such as *S. magna* and *S. antennata,* also presented middle-range population size and they may play a role in the transmission. Although the other species (*S. clydei, S. adleri* and *S. buxtoni*) can not be excluded, their involvement seems less likely due to their low abundance indoors and in peri-domestic habitats (Table 3). It is worth noting that the negative association between *S. buxtoni* captures and dog seroprevalence should be interpreted as an indirect effect, since *S. buxtoni* captures were negatively correlated with the captures of several other species (although not *S. dubia*).

In summary, several decades after the discovery of the Mont Rolland focus of canine leishmaniasis, the vector has not been identified yet, despite the important number of cases and the strong prevalence of serum positivity in humans and dogs [7]. Our data suggest that particular attention should be given to species of the genus *Sergentomyia* and particularly to the species *S. dubia* and *S. schwetzi*, which might be capable of transmitting canine leishmaniasis. This hypothesis will need to be more thoroughly evaluated. To validate this model, it is now important to carry out insect collections around infected dogs, to isolate the *Leishmania* parasites in the digestive tubes of phlebotomine sand flies by dissecting the females, to analyse the blood meals in order to determine on which host the phlebotomine sand flies had their meal, to detect by PCR amplification in female individuals the presence of *Leishmania* parasites and to identify the species.
